# Growth Performance and Meat Characteristics of the First Awassi–Rambouillet Callipyge Backcross

**DOI:** 10.3390/ani9080517

**Published:** 2019-08-01

**Authors:** Khaleel I. Jawasreh, Ahmad Hasen Alamareen, Mohammad Diya Obeidat, Pauline Y. Aad

**Affiliations:** 1 Department of Animal Production, Jordan University of Science and Technology (JUST), Irbid 22110, Jordan; 2 Livestock Research Directorate, National Agriculture Research Center (NARC), Albaqa’a 19381, Jordan; 3 Department of Sciences, Notre Dame University Louaize, Zouk Mosbeh 2207, Lebanon

**Keywords:** Awassi sheep, crossbreeding, gene introgression, growth performance, meat characteristics

## Abstract

**Simple Summary:**

The Awassi sheep breed is very resilient in the Mediterranean region but shows poor meat quality. The callipyge mutation enhances growth and meat characteristics, and as such we bred Awassi-Rambouillet Callipyge carrier rams with Awassi ewes and compared meat quality in the first backcross of Awassi-Rambouillet callipyge carriers (CRAW), non carrier Rambouillet Awassi (RAW), and pure Awassi (AW) ram lambs in a fattening trial for 94 days. CRAW lambs showed mostly similar growth potential and meat characteristics to RAW lambs. However, CRAW lambs showed higher growth potential as compared to AW lambs, and better meat characteristics in the shoulders and legs, but not in tenderness. Fat tail weight did not change in CRAW as compared to AW lambs. Therefore, the introgression of the callipyge mutation in Awassi lambs allows for improvement in growth and meat, without impacting the currently desirable traits of the fat tail. This research allows the improvement of meat production and return on investment in the local flocks of Awassi sheep.

**Abstract:**

The Awassi breed is desirable due to its resilient traits, but shows poor lean meat content on the carcass; the callipyge mutation may enhance growth and meat characteristics. The first backcross of callipyge Awassi–Rambouillet (CRAW) lambs was generated by mating Awassi ewes with heterozygous F1 (50 % Awassi and 50 % Rambouillet) rams for the callipyge mutation. A fattening trial with carriers of *CLPG* (CRAW), non-carriers (RAW), and Awassi (AW) ram lambs was conducted for 94 days and growth and meat characteristic parameters were recorded. Final body weight, average daily gain (ADG), feed conversion ratio, and dry matter intake, but not initial body weight, were greater in CRAW and RAW lambs as compared to AW lambs; hot and cold carcass, dressing percentage, and shoulder, rack, and loin weights were greater in CRAW vs. AW but not RAW lambs. Leg cuts were heavier in CRAW vs. both RAW and AW lambs; intermuscular and subcutaneous fat percentages were greater in CRAW as compared to AW but not RAW lambs. Non-carcass components showed kidney fat, kidney, liver and testes weights higher in CRAW lambs as compared to AW lambs, but these parameters were similar to those of RAW lambs. There were no differences in fat tail weight. Further, CRAW lambs had higher shear force and water holding capacity than RAW and AW lambs, while cooking loss was the lowest in CRAW lambs. In conclusion, the callipyge mutation with 25% Rambouillet genes can provide efficient improvements in growth and meat characteristics, with the exception of tenderness in Awassi sheep.

## 1. Introduction

Awassi, a multi-purpose fat-tailed sheep breed, is the predominant sheep breed in Jordan and the Middle East due to its adaption to the harsh environmental conditions of the region. Awassi sheep meat is the favored meat by Jordanian consumers, with high market prices and body and tail dimensions that are commercially important for trading lambs within local markets. In fact, Awassi lambs are preferable in Jordan regardless of whether they are pure- or cross-bred as long as they present a fat tail. However, the Awassi breed suffers from inferior carcass merit and poor leg muscling [1], and therefore the integration of the *CLPG* mutation, along with 25% Rambouillet genes, in breeding strategies has the potential to significantly improve lamb meat quality.

The callipyge phenotype (CLPG) presents generalized muscular hypertrophy, and was first described in Dorset sheep in Oklahoma [2]. The leg and loin muscle weights were significantly increased in sheep expressing this condition [3,4], but the expression of *CLPG* was absent in the diaphragm and some muscles of the shoulders [3]. The *CLPG* locus has been mapped to the telomeric region of ovine chromosome 18 [2], inherited in a non-Mendelian pattern termed polar overdominance. The callipyge phenotype is only expressed in heterozygous animals that inherit the mutation from their sire, i.e., the *^+M^/CLPG^P^* genotype, where the superscripts M and P refer to the maternal or paternal inheritance of the alleles, respectively, for both homozygous +M/+P and *CLPG^M^/CLPG^P^*. Animals that inherit the mutation from their mothers (*CLPG^M/+P^*) show normal muscle development [5].

CLPG lambs exhibit a normal muscle phenotype at birth, and the CLPG phenotype becomes apparent in the lambs at approximately 4 to 6 weeks postpartum [6]. Unlike the prenatally expressed double muscling gene mutation in cattle, postnatal CLPG development of muscling does not increase any risk of dystocia in sheep. The advantage of the callipyge phenotype, as described in several studies [3,4,6], lies in the larger longissimus loin eye areas, superior lean composition, higher leg scores and dressing percentages, and optimal feed efficiency. Koohmaraie et al. [4] reported that tenderness of CLPG longissimus muscle is the only trait that seems to be affected negatively by the *CLPG* gene.

This study is part of a long program from the Jordan University of Science and Technology to develop a desirable Awassi phenotype with improved meat characteristics, including feed efficiency, meat growth, and tenderness by the introgression of the callipyge mutation into Awassi sheep [7]. To our knowledge, this the first study to investigate the effect of 25% Rambouillet genes and the *CLPG* mutation on Awassi growth and meat characteristics. The objectives of this study were to evaluate the callipyge mutation effect on growth performance, as well as the carcass characteristics and meat quality of a callipyge Awassi–Rambouillet first back-cross, and compare them with the Awassi and Rambouillet cross.

## 2. Materials and Methods

All procedures used in this study were approved by the Animal Care and Use Committee at Jordan University of Science and Technology (JUST), Jordan (Nº16/3/3/534), in line with the international ethical guidelines of humane treatment of experimental animals (IACUC).

The callipyge mutation was introduced to the local Awassi sheep in 2011 by insemination with imported frozen semen of four Rambouillet homozygous rams for the *CLPG* gene from Utah University, USA. The first-generation callipyge Awassi rams (F1-CA, 50% Callipyge and 50% Awassi) characterized by Jawasreh et al. [7,8], were used for mating the Awassi ewes to produce the first backcross of callipyge Awassi–Rambouillet lambs (CRAW), resulting in rams carrying 25% Rambouillet and 75% Awassi genes. Further, mating Awassi ewes with F1-CA sires heterozygous for the *CLPG* gene resulted in a Rambouillet–Awassi ram lambs without the *CLPG* gene (RAW), composed of 25% Rambouillet and 75% Awassi genes. Awassi lambs (AW) were found in the same station and were of the same age and weight. The CRAW ram lambs were further examined to determine carrier lambs of the *CLPG* gene by PCR-RFLP technique according to Freking et al. [9], and were later evaluated phonetically for the existence of the CLPG phenotype at the age four to six weeks.

Blood samples were collected from jugular vein of the 110 first backcross lambs (25% Rambouillet and 75% Awassi, aged 3 weeks) that resulted from mating of the heterozygous callipyge-Awassi ram (50% Awassi and 50% Rambouillet) with Awassi ewes. Samples were collected in vacationer tubes containing EDTA and stored at 4 °C.

DNA was isolated from the blood samples using a DNA isolation kit (Omega Bio-Tek Inc., Norcross, GA, USA). The quantity and quality of genomic DNA were ascertained by ultraviolet light and 1.5% Agarose Gel electrophoresis.

The DNA amplification of the *CLPG* gene was achieved by polymerase chain reaction (PCR). Two primer pairs (5’-TGA AAA CGT GAA CCC AGA AGC-3’ (forward) and 5’-GTC CTA AAT AGG TCC TCT CG-3’ (reverse))) that targeted a fragment of 426 bp as described by Freking et al. [9] were used for the identification of the *CLPG* gene. The PCR amplification reaction solution was performed in total volume of 25 μL containing 2 μL (200 ng) DNA, 4.5 μL nuclease free water, 12.5 µl of GoTaq^®^ (Promega Corporation, Madison, WI, USA) Green Master Mix (2×), 2 µL MgCl_2_, and 2 μL (0.8 µM) of each primer. The PCR cycling condition was a preliminary denaturizing at 95 °C for 3 min, followed by 1 cycle of denaturing at 95 °C for 30 s, annealing at 59 °C for 30 s, and extension at 72 °C for 40 s, followed by 35 cycles and 10 min at 72 °C as a final extension. The PCR reactions were performed on a LifePro Thermal Cycler. The PCR products of 426 bp were confirmed by resolving on 1.5% agarose containing (ethidium bromide) in parallel with a 100-bp DNA ladder. Gel electrophoresis was carried out at a constant voltage of 120 V for 45 min.

The amplified fragment of *CLPG* (426 bp) was digested by the restriction endonuclease BsmFI (Fermentas, Waltham, MA, USA). Digestion was conducted at 37 °C for 13 h and in a 25-µL reaction solution including 2 µL of nuclease free water, 6 µL of buffer, 1 µL Bovine Serum Albumin, 1 µL of BsmFI, and 15 µL of PCR product. The digested fragments were separated by electrophoresis on 3% agarose gel stained with ethidium bromide and the gel was analyzed in the UV rays.

The fattening trial for the ram lambs was conducted at Jordan University of Science and Technology Center for Extension and Research using 24 randomly selected unrelated ram lambs (weaned at 60 ± 2 days old). In order to avoid weaning stress, the lambs underwent two weeks of adaptation and reared in 1.5 × 1.75 m^2^ individual pens. The three genotype groups were evaluated in this trial with eight callipyge Awassi–Rambouillet ram (CRAW) lambs, eight non-CLPG Rambouillet–Awassi ram (RAW) lambs, and eight Awassi ram (AW) lambs. All lambs were introduced slowly to ad libitum access to water and a diet containing 16% crude protein (CP) and 2.0 Mcal metabolizable energy/kg for a period of 94 days [10]. The total mixed ration contained 15% soybean, 61.4% barley, 21% wheat straw, 1.5% salt, 0.1% limestone, 0.1% minerals, and 0.1% vitamins. Feed and orts were weighed daily for the calculation of feed intake and efficiency, while lamb live weights were recorded weekly.

For the purpose of carcass evaluation and meat quality measurements, all ram lambs were slaughtered at the same time at the end of the fattening period, after 12 h of fasting with free access to water. The lambs were slaughtered using a standard slaughter procedure as described by Abdullah et al. [11]. Briefly, carcasses were divided into five parts namely shoulder, rack, loin and leg cuts, and fat tail [12], and their weights recorded. The rib-eye area, fat depths, tissue depth (GR), rib fat depth (J), eye muscle width (A), depth (B), eye muscle area, fat depth (C), shoulder fat depth (S2), and leg fat depth (L3) in addition to longissimus muscles weight were measured on chilled (at 5 °C for 24 h) cuts [11]. Each major cut was separated into right and left sides using an electrical saw. The right side of each cut was sealed in a plastic bag and frozen at −20 °C for later analysis while the left leg was dissected to determine its muscle, bone, subcutaneous fat, and inter muscular fat components as indicators of muscularity of the whole carcass. Longissimus muscles were excised from the right side of loin cuts, cleaned from the subcutaneous fat, vacuum-packaged, and frozen at −20 °C for later analysis. Meat quality measurements were conducted on cooked meat samples as described by Abdullah and Qudsieh [13], including Warner–Bratzler shear force values, water holding capacity, cooking loss, and color coordinates (L*, a*, and b*).

Data were analyzed using the mixed general linear model ANCOVA using the mixed procedure of SAS (SAS Institute, Cary, NC, USA). The genotype of the animals was inserted as fixed effect in the model. The initial weight was inserted as a covariate for the final body weight, fasting live weight, hot carcass weight, cold carcass weight, dressing percentage and average daily gain. Dry matter intake, feed conversion ratio, carcass cut, dissected leg, non-carcass components, and meat quality were also analyzed using the mixed procedure of SAS without including covariate factors in the model. The sires of the animals were inserted as Random effect for all studied traits. Differences among means were tested using the LSD-test procedure by the inclusion of the PDIFF option in the LSMEANS statement and values presented as least square means (LSMeans) ± SE of the mean (SEM).

## 3. Results

One hundred and ten newborn first backcross lambs that resulted from mating of the heterozygous callipyge Awassi ram (50% Awassi and 50% Rambouillet) with Awassi ewes were examined to determine carrier lambs of *CLPG* gene by the polymerase chain reaction followed by restriction fragment length polymorphisms (PCR-RFLP) technique.

**Figure 1:**
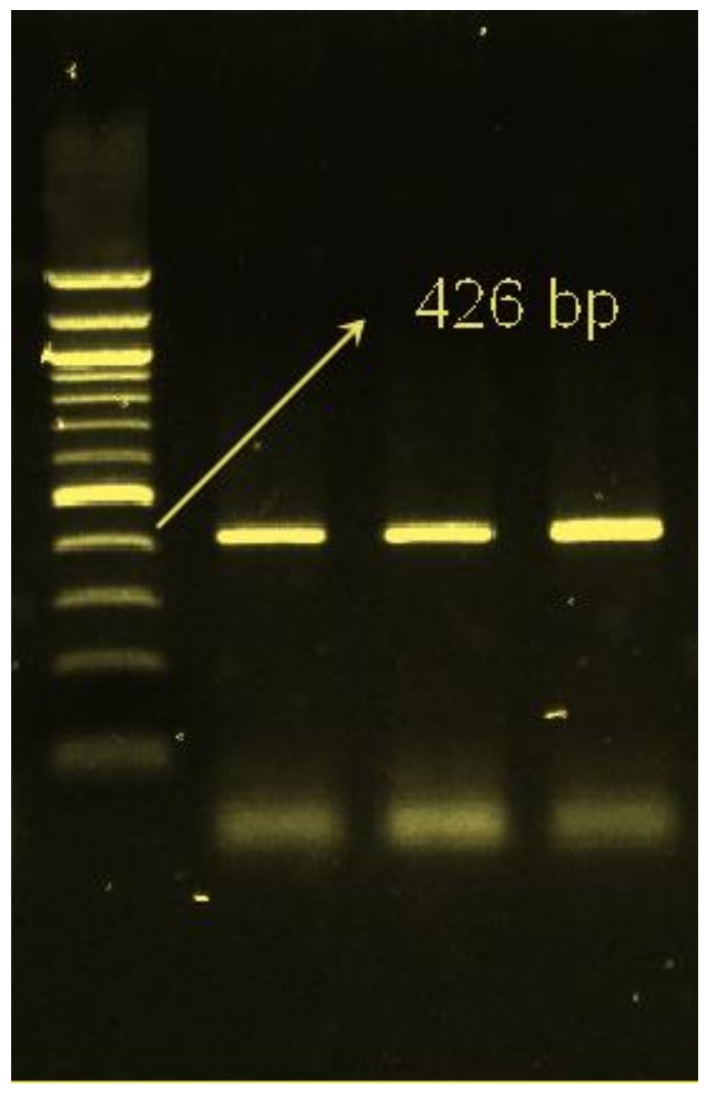
PCR product of the *CLPG* mutation (426 bp) visualized using 1.5% agarose.

**Figure 2:**
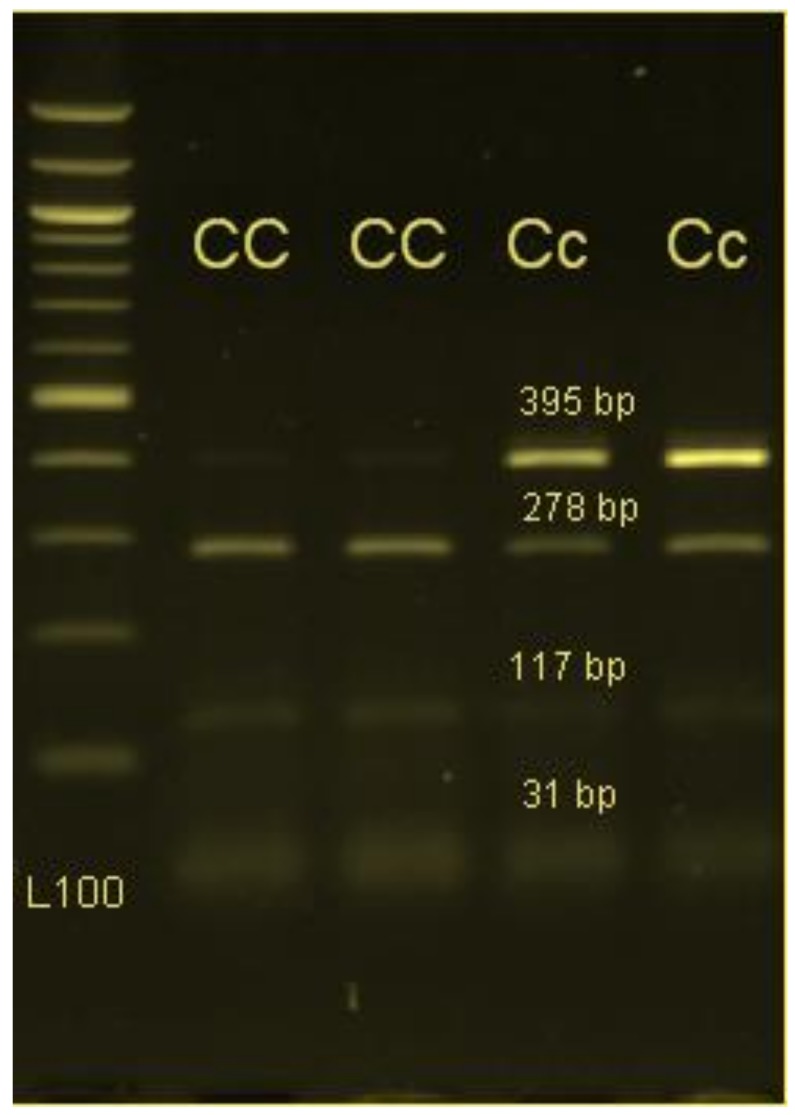
PCR-RFLP results for *CLPG* gene by BsmFI restriction enzyme on 2% agarose gel. PCR products produced segments of 395 bp and 31 bp for mutant allele c and 278 bp, 117 bp, and 31 bp for wild type allele C.

The amplification of the targeted BsmFI *CLPG* gene show fragments of size 426 bp (Figure 1). Genotyping of *CLPG* gene was conducted by the digesting the PCR product by restriction BsmFI enzyme. The digestion of the PCR products produced segments of 395 bp and 31 bp for mutant allele c and 278 bp, 117 bp, and 31 bp for wild type allele C (Figure 2). As expected and according to Mendelian inheritance, half of the lambs (55 lambs) were carriers for the *CLPG* mutation since the rams were of the heterozygous genotype.

At the beginning of the fattening trial, there was no significant difference in the initial body weight among all genotypes. The CRAW lambs did not differ (*p* > 0.05) in final body weight from RAW lambs but were heavier (*p* < 0.05) than AW lambs. Furthermore, the callipyge mutation and 25% Rambouillet genes had a positive effect on the average daily gain (ADG) of the lambs for the whole trial period. The CRAW lambs had higher (*p* < 0.05) ADG than AW lambs (0.326 vs. 0.177 kg/d), whereas the ADG of CRAW lambs was comparable to that of RAW lambs (0.326 vs. 0.298 kg/d; *p* > 0.05). CRAW and RAW consumed significantly more feed than AW during the fattening period, resulting in lower (*p* < 0.05) feed conversion ratio in CRAW as compared to AW lambs, but not RAW lambs, as shown in Table 1.

Hot and cold carcass weights were heavier (*p* < 0.05) for the CRAW lambs when compared to AW lambs but similar to RAW lambs (Table 2). Carcasses from CRAW were higher (*p* < 0.05) by 4% in dressing percentage than AW carcasses but similar to RAW lambs (*p* > 0.05). The legs weight was heavier for the CRAW lambs when compared to RAW or AW lambs (10.1 vs. 8.3 and 6.3 kg respectively; *p* < 0.05). The weight of shoulders racks and loins were higher (*p* < 0.05) in CRAW than AW, but not RAW (Table 2), with no difference (*p* > 0.05) in fat tail weight between all lambs.

Weights of non-carcass components are presented in Table 2. Some non-carcass component weights, including liver, kidney, kidney fat, and testes weight were similar between RAW and CRAW but higher (*p* < 0.05) in CRAW compared to AW lambs, while there were no significant differences (*p* > 0.05) in heart, spleen, mesenteric fat, lungs, and trachea weights among the three genotypes.

The effects of genotype on muscle fat and bone weights are shown in Table 3. The longissimus muscle weight was 36% heavier (*p* < 0.001) in CRAW than RAW and 85% heavier than AW. Furthermore, total lean weight was 66% heavier in CRAW than AW and 29% heavier than RAW. Intermuscular fat, subcutaneous fat and total bone in leg muscle were higher in CRAW than in AW, while there were no differences (*p* > 0.05) in intermuscular fat, subcutaneous fat, and total bone between the CRAW and RAW lambs.

The effect of the CLPG and Rambouillet phenotypes on longissimus muscle linear dimensions and fat measurements is presented in Table 3. The eye muscle area of CRAW was significantly larger (*p* < 0.05) than RAW and AW (25.5 vs. 19.1, 15.1 cm^2^, respectively). Similarly, longissimus muscle width and depth were larger (*p* < 0.05) for CRAW compared to RAW or AW lambs. Some linear fat measurements such as tissue depth (GR), leg fat thickness (L3), and shoulder fat thickness (S2) were also higher (*p* < 0.05) in CRAW compared to AW but similar to RAW lambs. Finally, no differences (*p* > 0.05) were observed in rib fat depth in all genotypes, while fat depth in longissimus dorsi was not different (*p* > 0.05) between RAW and AW.

Meat quality characteristics are shown in Table 4, where shear force measurements of CRAW were higher (*p* < 0.05) than both RAW and AW (7.2 vs. 4.2, 3.2 kg, respectively). Cooking loss for CRAW was lower (*p* > 0.05) than AW, but similar (*p* > 0.10) to RAW lambs, and the water holding capacity was higher (*p* > 0.05) in CRAW compared to RAW and AW meats (26.6 vs. 22.0, 19.5%, respectively). Furthermore, no differences (*p* > 0.05) in meat pH or color coordinates (L*, a* and b*) were observed in all evaluated meat cuts.

In a nutshell, CRAW and RAW were generally superior to AW in meat performance, but similar amongst each other except for leg weight, water holding capacity, shear force measurement and fat content. Altogether our results show a slight advantage to the introgression of the callipyge gene than just Rambouillet genotype in our rearing system, where leaner meat is becoming more desirable for human consumption in Jordan and the Mediterranean region.

## 4. Discussion

In this study, we expanded the findings from our previous work [7,8,14], where we introduced the CLPG in Awassi flocks and evaluated the effects of this CLPG in F1 (callipyge Awassi–Rambouillet lambs) to highlight the specific combined performance and meat characteristics traits of CLPG and 25% Rambouillet as compared to Awassi.

The body weight increase in CRAW lambs was in accordance with the results of Srinivasan [15] where slaughter weights of the Callipyge lambs were higher (*p* < 0.05) than normal lambs (59 vs. 55 kg). No differences between the CRAW and RAW groups were observed in growth performance, that were in agreement with previous reports where little or no effect of the CLPG on lamb growth was induced when compared to their normal siblings [3,16,17,18]. Jackson et al. [6] and Al-Dabeeb [19] both found that lambs expressing the callipyge muscle phenotype consumed less feed per kg of live weight than normal half-sibling lambs. In contrast, callipyge Awassi lambs had a lower feed conversion ratio compared to normal half-siblings [6]. Further, CLPG lambs showed heavier hot and cold carcass weights when compared to non-carrier lambs [6,9,20,21] where the dressing percentage ranged from 2% [4] to 5% [22], and contradictory findings regarding the increase in dressing percent due to an increase in muscle mass were reported by Jackson et al. [22] but not Koohmaraie et al. [4]. Previous reports also showed that the dressing percent was also influenced by fat content [4], gut-fill or lower intestinal weight [4,22], and wool as compared with non-carrier lambs.

Similar to our findings for the CLPG for Rambouillet crosses, higher shoulder, leg, rack, and loin cut weights in CLPG carcasses compared to non-carrier lamb carcasses were reported by Jawasreh et al. [8], Abdulkhaliq et al. [16,17], Goodson et al. [23], and Field et al. [21]. It is important to note that the lambs in this study resulted from the first backcross of callipyge Awassi with Awassi lambs, carrying 25% Rambouillet and 75% Awassi genes. As a result, a possible reversion of the Awassi fat tail phenotype closer to that of Awassi sheep, a desirable characteristic by farmers and consumers, was observed in this study. This was in contrast to greater fat tail weight in F1 Awassi callipyge rams as reported by Jawasreh et al. [8] using the F1 callipyge/Rambouillet–Awassi cross (50% Awassi and 50% Rambouillet). Perhaps the 25% meat-type Rambouillet genes, in addition to the *CLPG* gene, may be responsible for such an increase in growth performance, and this appears in the comparisons made between CRAW and RAW lambs, where these two groups did not differ in the majority of enhanced traits.

The muscle fat and bone weights in CRAW lambs were higher than in AW lambs, but not in RAW lambs, in agreement with previous work where callipyge rams were heavier compared to the Awassi lambs or non-carriers lambs [17,20,21,23,24]. Specifically, greater longissimus muscle weights were reported to be 27% [25], 32% [26], 39% [23], 42% [27], and even 51% [22] greater in *CLPG* gene-carrying vs. non-carrying rams. Earlier, our lab (unpublished data) found that total leg muscle weight was significantly higher in the callipyge Awassi (50% Rambouillet) compared to the Awassi lambs, in agreement with our results. Published results [3,4,17,23,25,28,29] indicated that individual muscle weights in the hind limbs were increased in lambs carrying the *CLPG* gene compared to non-carrier animals. However, opposite to our findings, Jawasreh et al. [8] reported lower intermuscular fat and subcutaneous fat in leg muscle in callipyge Awassi (50% Rambouillet) compared to AW lambs. This lower fat concentration in callipyge lambs appears to be due to less intramuscular fat in this study. Jackson et al. [22] reported that the difference in total carcass fat between the two phenotypes was mainly due to a lower amount of intermuscular and intramuscular fat in the carcasses. Goodson et al. [23] indicated that lambs expressing the callipyge mutation had total fat as a percentage compared to carcass side weight than lambs not expressing the callipyge mutation (19.7 vs. 29.0%). Furthermore, Jawasreh et al. [8] found a non-significant difference in total bone weight between callipyge Awassi (50% Rambouillet) and Awassi lambs, and Goodson et al. [23] found that the percentage of bone was similar between the two phenotypes. However, Jackson et al. [22] found that Callipyge lambs had lower percentage of bone as compared to normal lambs.

Differences in muscle weights in CLPG lambs observed in the leg and loin cuts in our study may result from an increased expression of the callipyge mutation [6]. Stockdale [30] linked the increased muscle mass to the increased satellite cell proliferation and a capacity to synthesize protein and reduce protein degradation in callipyge muscles. Carpenter et al. [24] concluded that the large increase in muscle mass (semitendinosus, longissimus, and gluteus medius) in CLPG lambs was strongly associated with changes in the fast twitch glycolytic (FG) fibers, and that the enlargement of muscles in callipyge-expressing animals was primarily due to myofiber hypertrophy. Carpenter et al. [24] also found that hypertrophy-responsive muscles from callipyge lambs were of larger average diameters in FG and fast oxidative glycolytic (FOG) muscle fibers, but smaller average diameter in slow twitch oxidative (SO) fibers when compared to normal lambs. Lorenzen et al. [31] reported the increase in protein mass and the reduction in degraded protein in callipyge muscles (longissimus and biceps femoris) being predominantly due to an absolute increase in tissue weight.

Similar to previous reports, some non-carcass components were higher in this study in callipyge Awassi (25% Rambouillet) lambs as compared to Awassi lambs, while spleen weight was not different [4]. Jackson et al. [22] reported no differences in internal organ weights like spleen, lungs and mesentery fat between lambs expressing or not the callipyge phenotype, while Koohmaraie et al. [4] found that CLPG lambs had lighter internal organ weights in terms of lungs, liver, and kidney than normal lambs. Abdulkhaliq et al. [16,17], Goodson et al. [23], Freking et al. (1998b) [18], and Srinivasan [15] all reported that callipyge lambs had lower liver, kidney and pelvic fat weights than normal lambs. Further, the correlation between testicular weight and body weight was described to be significantly high and positive in Awassi sheep [32,33], with no indication on this correlation in *CLPG* gene carrying lambs.

The effect of the CLPG phenotype combined with 25% of Rambouillet genes on longissimus muscle linear dimensions and fat measurements were apparent in this study. These were in agreement with Jawasreh et al. [8] where loin muscle area was larger in callipyge Awassi (50% Awassi and 50% Rambouillet) compared to Awassi lambs (30.97 vs. 16.77 cm^2^). Further, an increase in the longissimus muscle area of the callipyge lambs when compared to non-carriers lamb were reported [4,5,17,20,21,23,24] to be larger in width and depth than in AW lambs. Interestingly, all linear fat measurements (higher in CRAW than AW) in this study were opposite to previous reports [4,16,18,20,21,22,23] where CLPG lambs showed less fat thickness compared to the normal lambs. It is crucial to note that these effects may be affected by both the 25% Rambouillet genes in addition to the *CLPG* gene.

The higher shear force measurements in the muscles of the CLPG by Rambouillet cross are in consistence with previous reports [4,8,17,20,21,23,34]. The increased toughness of CLPG meat has been attributed to higher calpastatin activity, resulting in decreased protein degradation [4,23,34]. Goodson et al. [23] indicated that the increased toughness of the longissimus thoracis et lumborum in the CLPG lambs was apparently due to lower myofibrillar component rather than the connective tissue component of the muscle. Koohmaraie et al. [4] suggested that the reduction rate of protein degradation and a higher capacity for protein synthesis are consequences of the callipyge condition, which in turn are associated with lower meat tenderness resulting from reduced rate and extent of postmortem meat proteolysis. Abdulkhaliq et al. [17] also suggested that the method of cooking may play a major role in the gene effect on meat tenderness.

Cooking loss in this study was lower in CRAW than AW, in accordance to some previous reports [12,35]. Higher water holding capacities in CRAW indicate a higher moisture content of uncooked loin muscle for CLPG lambs [17]. Jawasreh et al. [8] found no difference in water holding capacity between CLPG lambs (50% Rambouillet and 50% Awassi) and normal lambs. Similar to our results, no difference was reported between all genotypes in the meat pH [4], although changes in the proportion and size of muscle fibers were noted with an increase in the proportion of the glycolytic fibers associated with CLPG [4]. However, these results are in contrast to those of Jawasreh et al. [8] and Goodson et al. [23] who found meat pH to be lower in CLPG lambs than normal lambs. Color coordinates (L*, a* and b*) did not significantly differ between all meats in this study, similar to the results of Koohmaraie et al. [4].

It is crucial to point out that although most of our results are consistent with the findings of several studies that report the stated effects of *CLPG* gene introgression in the Awassi breed, the influence of Rambouillet genes introduced from the first backcross of callipyge Awassi–Rambouillet lambs should not go unnoticed; this observation is extrapolated from the similarities of results between the CRAW and RAW groups, where the latter did exhibit some exclusively favorable improvements. It would be interesting to conduct further future investigations using Rambouillet breeds as additional controls alongside the Awassi breed. Nonetheless, our study significantly points out the superior qualities of the newly generated genetic group following both manipulations.

## 5. Conclusions

The growth performance of the Awassi sheep has been improved through the introgression of 25% Rambouillet sheep and the callipyge mutation in Awassi sheep. The *CLPG* gene effects observed in the F1 backcross of this study improved growth characteristics and meat type, with the exception of tenderness, which was better than that of the first filial Rambouillet callipyge Awassi sheep, while safeguarding the desirable Awassi traits. Further research on the implementation of successful breeding strategies for the maintenance of the new backcross with the introgression of the new qualities requires further investigation, namely in order to determine the appropriate mix of Rambouillet/Awassi breeds in the presence or absence of the *CLPG* mutation to improve meat tenderness in addition to the other growth and meat characteristics. The synergetic effects of the CLPG and Rambouillet genes can be used in structured mating systems to make dramatic improvements in total lean, feed efficiency, and carcass composition of Awassi sheep.

## Figures and Tables

**Table 1:** Growth performance of first backcross callipyge Awassi–Rambouillet (CRAW), Rambouillet–Awassi (RAW), and Awassi (AW) lambs.

Trait	Genotype *			Covariate **
	CRAW	RAW	AW	
Initial weight (kg)	22.80 ± 1.43	20.90 ± 1.43	19.30 ± 1.43	
Final body weight (kg)	50.80 ^a^ ± 1.72	48.20 ^a^ ± 1.76	37.10 ^b^ ± 1.73	0.0005
Dry matter intake (kg)	115.70 ^a^ ± 5.27	109.50 ^a^ ± 5.27	83.60 ^b^ ± 5.27	
ADG (kg)	0.33 ^a^ ± 0.02	0.30 ^a^ ± 0.02	0.18 ^b^ ± 0.02	0.762
FCR (kg feed/kg gain)	3.90 ^a^ ± 0.25	4.10 ^a^ ± 0.25	5.40 ^b^ ± 0.25	

* Values presented as least square means ± SEM. ADG: average daily gain, FCR: feed conversion ratio. ^a,b,c^ Means within the same row with different superscripts differ according to the indicated level of significance (*p* < 0.05). ** Significant covariate included in the model. Initial weight was inserted as covariate to account for initial weight differences and adjust subsequent weights and measurements. *p* ≤ 0.05 was considered significant.

**Table 2:** Carcass and non-carcass components of the first backcross callipyge Awassi–Rambouillet (CRAW), Rambouillet–Awassi (RAW), and Awassi (AW) lambs.

Weight (kg)	Genotype *			Covariate **
	CRAW	RAW	AW	
Carcass evaluation				
Fasting body	48.5 ^a^ ±1.96	45.0 ^a^ ± 1.63	35.9 ^b^ ± 1.68	0.0008
Hot carcass	26.4 ^a^ ± 1.00	24.0 ^a^ ± 0.97	18.0 ^b^ ± 1.00	0.0001
Cold carcass	25.6 ^a^ ± 0.98	23.3 ^a^ ± 0.95	17.5 ^b^ ± 0.98	0.0001
Legs	10.1 ^a^ ± 0.64	8.3 ^b^ ± 0.59	6.3 ^c^ ± 0.94	
Loins	2.8 ^a^ ± 0.18	2.5 ^a^ ± 0.17	1.5 ^b^ ± 0.27	
Shoulders	10.4 ^a^ ± 0.61	9.3 ^a^ ± 0.56	7.2 ^b^ ± 0.95	
Racks	2.6 ^a^ ± 0.17	2.3 ^a^ ± 0.17	1.4 ^b^ ± 0.17	
Fat tail	1.2 ± 0.27	1.1 ± 0.25	1.5 ± 0.39	
Dressing percent (%)	54.8 ^a^ ± 1.01	53.5 ^a^ ± 0.90	50.7 ^b^ ± 1.45	0.0029
Organ weight (kg)				
Heart	0.270 ± 0.03	0.260 ± 0.02	0.205 ± 0.03	
Liver	0.720 ^a^ ± 0.04	0.660 ^a,b^ ± 0.04	0.550 ^b^ ± 0.04	
Spleen	0.080 ± 0.01	0.082 ± 0.01	0.060 ± 0.01	
Kidney	0.126 ^a^ ± 0.01	0.124 ^a^ ± 0.01	0.090 ^b^ ± 0.01	
Kidney fat	0.260 ^a^ ± 0.04	0.238 ^a^ ± 0.04	0.107 ^b^ ± 0.06	
Mesenteric fat	0.470 ± 0.06	0.481 ± 0.06	0.460 ± 0.10	
Lungs and trachea	0.583 ± 0.04	0.608 ± 0.04	0.526 ± 0.04	
Testes	0.270 ^a^ ± 0.02	0.251 ^a^ ± 0.02	0.149 ^b^ ± 0.03	

* Values presented as least square means ± SEM. ^a,b,c^ Means within the same row with different superscripts differ according to the indicated level of significance (*p* < 0.05). ** Significant covariate included in the model (initial weight was inserted as covariate to account for initial weight differences and adjust subsequent weights and measurements), *p* ≤ 0.05 considered significant.

**Table 3:** Means (±SEM) dissected leg, loin cuts, longissimus muscle, and fat measurements of the first backcross callipyge Awassi–Rambouillet (CRAW), Rambouillet-Awassi (RAW), and Awassi (AW) lambs.

Traits	Genotype *		
	CRAW	RAW	AW
Carcass weight (kg)			
Longissimus muscle	0.363 ^a^ ± 0.02	0.267 ^b^ ± 0.02	0.196 ^c^ ± 0.04
Leg	5.051 ^a^ ± 0.32	4.102 ^b^ ± 0.30	3.075 ^c^ ± 0.48
Intermuscular fat	0.140 ^a^ ± 0.02	0.114 ^a^ ± 0.02	0.079 ^b^ ± 0.02
Subcutaneous fat	0.688 ^a^ ± 0.07	0.561 ^a^ ± 0.07	0.349 ^b^ ± 0.07
Total lean	3.021 ^a^ ± 0.19	2.336 ^b^ ± 0.18	1.821 ^c^ ± 0.30
Total bone	0.861 ^a^ ± 0.04	0.765 ^a,b^ ± 0.04	0.664 ^b^ ± 0.04
Eye muscle			
Width (A) (mm)	73.4 ^a^ ± 1.54	67.4 ^b^ ± 1.54	59.9 ^c^ ± 1.54
Depth (B) (mm)	39.7 ^a^ ± 1.46	32.2 ^b^ ± 1.39	26.0 ^c^ ± 1.99
Area (cm^2^)	25.5 ^a^ ± 1.34	19.1 ^b^ ± 1.24	15.1 ^c^ ± 2.01
Fat			
Depth (C) (mm)	4.9 ^a^ ± 0.72	4.0 ^a,b^ ± 0.72	2.1 ^b^ ± 0.72
Thickness (L3) (mm)	10.3 ^a^ ± 1.17	10.3 ^a^ ± 1.17	6.4 ^b^ ± 1.17
Thickness (S2) (mm)	6.38 ^a^ ± 0.75	6.29 ^a^ ± 0.75	3.75 ^b^ ± 0.75
Tissue depth (GR) (mm)	20.3 ^a^ ± 1.44	19.4 ^a^ ± 1.44	13.7 ^b^ ± 1.44
Rib fat depth (J) (mm)	8.62 ± 0.90	7.53 ± 0.87	6.48 ± 1.12

* Values presented as least square means ± SEM. ^a,b,c^ Means within the same row with different superscripts differ according to the indicated level of significance (*p* < 0.05).

**Table 4:** Means (±SEM) meat quality characteristics of the first backcross callipyge Awassi-Rambouillet (CRAW), Rambouillet–Awassi (RAW), and Awassi (AW) lambs.

Traits	Genotype *		
	CRAW	RAW	AW
Cooking loss (%)	41.3 ^a^ ± 0.48	42.2 ^a,b^ ± 0.48	43.1 ^b^ ± 0.48
Water holding capacity (%)	26.6 ^a^ ± 2.31	22.0 ^b^ ± 2.10	19.5 ^b^ ± 3.66
Shear force (kg/cm^3^)	7.28 ^a^ ± 0.51	4.25 ^b^ ± 0.51	3.22 ^b^ ± 0.51
pH	5.87 ± 0.38	5.80 ± 0.38	5.80 ± 0.38
Color			
a* (redness)	2.99 ± 19.7	2.72 ± 19.7	3.14 ± 19.7
b* (yellowness)	17.9 ± 68.9	18.1 ± 63.1	17.5 ± 106.4
L* (lightness)	35.5 ± 1.04	35.7 ± 1.04	35.3 ± 1.04

* Values presented as least square means ± SEM. ^a,b,c^ Means within the same row with different superscripts differ according to the indicated level of significance (*p* < 0.05).

## References

[1] Goot H., Bor A., Hasdai H., Zenou A., Gootwine E., Elsen J.M., Bodin L., Thimonier J. (1991). Body and carcass composition of Awassi, Assaf, Booroola × Awassi and Booroola × Assaf ram lambs. Major Genes for Reproduction in Sheep.

[2] Cockett N.E., Jackson S.P., Shay T.L., Nielsen D., Moore S.S., Steel M.R., Barendse W., Green R.D., Georges M. (1994). Chromosomal localization of the callipyge gene in sheep (*Ovis aries*) using bovine DNA markers. Proc. Natl. Acad. Sci. USA.

[3] Jackson S.P., Miller M.F., Green R.D. (1997). Phenotypic characterization of Rambouillet sheep expression the callipyge gene: III. Muscle weights and muscle weight distribution. J. Anim. Sci..

[4] Koohmaraie M., Shackelford S.D., Wheeler T.L., Lonergan S.M., Doumit M.E. (1995). A muscle hypertrophy condition in lamb (Callipyge): Characterization of effects on muscle growth and meat quality traits. J. Anim. Sci..

[5] Cockett N.E., Jackson S.P., Shay T.L., Farnir F., Berghmans S., Snowder G.D., Nielsen D.M., Georges M. (1996). Polar overdominance at the ovine callipyge locus. Science.

[6] Jackson S.P., Green R.D., Miller M.F. (1997). Phenotypic characterization of Rambouillet sheep expressing the callipyge gene: I. Inheritance of the condition and production characteristics. J. Anim. Sci..

[7] Jawasreh K.I.Z., Al-Amareen A.H., Aad P.Y. (2019). Growth performance and meat characteristics of the first filial Awassi Rambouillet callipyge ram lambs. Vet. World.

[8] Jawasreh K.I.Z., Abdullah A.Y., Talafha H.M., Ababneh H.S., Hijazeen F.A. Callipyge-Awassi Growth and Meat characteristics in comparison to the Pure Awassi sheep. Proceedings of the International Mesopotamia Agriculture Congress.

[9] Freking B.A., Keele J.W., Beattie C.W., Kappes S.M., Smith T.P., Sonstegard T.S., Nielsen M.K., Leymaster K.A. (1998). Evaluation of the ovine callipyge locus: I. Relative chromosomal position and gene action. J. Anim. Sci..

[10] National Research Council (1985). Designing Foods: Animal Product Options in the Market Place.

[11] Abdullah A.Y., Purchas R.W., Davies A.S. (1998). Patterns of change with growth for muscularity and other composition characteristics of Southdown rams selected for high and low back depth. N. Z. J. Agric. Res..

[12] Abdullah A.Y., Musallam H.S. (2007). Effect of different levels of energy on carcass composition and meat quality of male black goats kids. Livest. Sci..

[13] Abdullah A.Y., Qudsieh R.I. (2009). Effect of slaughter weight and aging time on the quality of meat from Awassi ram lambs. Meat Sci..

[14] Jawasreh K., Boettcher P.J., Stella A. (2016). Genome-wide association scan suggests basis for microtia in Awassi sheep. Anim. Genet..

[15] Haribaskar S. (1997). Effect of Callipyge Gene on Lamb Growth, Carcass Characteristics, and Meat Quality.

[16] Abdulkhaliq A.M., Meyer H.H., Busboom J.R., Thompson J.M. (2007). Growth, carcass and cooked meat characteristics of lambs sired by Dorset rams heterozygous for the Callipyge gene and Suffolk and Texel rams. Small Rumin. Res..

[17] Abdulkhaliq A.M., Meyer H.H., Thompson J.M., Holmes Z.A., Forsberg N.E., Davis S.L. (2002). Callipyge gene effects on lamb growth, carcass traits, muscle weights and meat characteristics. Small Rumin. Res..

[18] Freking B.A., Keele J.W., Nielsen M.K., Leymaster K.A. (1998). Evaluation of the ovine callipyge locus: II. Genotypic effects on growth, slaughter, and carcass traits. J. Anim. Sci..

[19] Al-Dabeeb S. (2000). Effect of Nutrition on Muscle Protein Turnover in Callipyge and Normal Lambs.

[20] Everts A.K., Wulf D.M., Wheeler T.L., Everts A.J., Weaver A.D., Daniel J.A. (2010). Enhancement technology improves palatability of normal and callipyge lambs. J. Anim. Sci..

[21] Field R.A., McCormick R.J., Brown D.R., Hinds F.C., Snowder G.D. (1996). Collagen crosslinks in longissimus muscle from lambs expressing the callipyge gene. J. Anim. Sci..

[22] Jackson S.P., Miller M.F., Green R.D. (1997). Phenotypic characterization of Rambouillet sheep expressing the Callipyge gene: II. Carcass characteristics and retail yield. J. Anim. Sci..

[23] Goodson K.J., Miller R.K., Savell J.W. (2001). Carcass traits, muscle characteristics, and palatability attributes of lambs expressing the callipyge phenotype. Meat Sci..

[24] Carpenter C.E., Rice O.D., Cockett N.E., Snowder G.D. (1996). Histology and composition of muscles from normal and callipyge lambs. J. Anim. Sci..

[25] Delgado E.F., Geesink G.H., Marchello J.A., Goll D.E., Koohmaraie M. (2001). The calpain system in three muscles of normal and callipyge sheep. J. Anim. Sci..

[26] Koohmaraie M., Shackelford S.D., Wheeler T.L. (1996). Effects of a beta-adrenergic agonist (L-644,969) and male sex condition on muscle growth and meat quality of callipyge lambs. J. Anim. Sci..

[27] Duckett S.K., Snowder G.D., Cockett N.E. (2000). Effect of the callipyge gene on muscle growth, calpastatin activity, and tenderness of three muscles across the growth curve. J. Anim. Sci..

[28] Duckett S.K., Klein T.A., Leckie R.K., Thorngate J.H., Busboom J.R., Snowder G.D. (1998). Effect of freezing on calpastatin activity and tenderness of callipyge lamb. J. Anim. Sci..

[29] Gootwine E., Zenou A., Bor A., Yossafi S., Rosov A., Pollott G.E. (2002). Introgression of the callipyge mutation into the Assaf fat tail breed. Options Méd..

[30] Stockdale F.E. (1992). Myogenic cell lineages. Dev. Biol..

[31] Lorenzen C.L., Koohmaraie M., Shackelford S.D., Jahoor F., Freetly H.C., Wheeler T.L., Savell J.W., Fiorotto M.L. (2000). Protein kinetics in callipyge lambs. J. Anim. Sci..

[32] Salhab S.A., Zarkawi M., Wardeh M.F., Al-Masri M.R., Kassem R. (2001). Development of testicular dimensions and size, and their relationship to age, body weight and parental size in growing Awassi ram lambs. Small Rumin. Res..

[33] Abdullah A.Y., Qudsieh R.I. (2008). Carcass characteristics of Awassi ram lambs slaughtered at different weights. Livest. Sci..

[34] Kerth C.R., Jackson S.P., Ramsey C.B., Miller M.F. (2003). Characterization and consumer acceptance of three muscles from Hampshire× Rambouillet cross sheep expressing the callipyge phenotype. J. Anim. Sci..

[35] Shackelford S.D., Wheeler T.L., Koohmaraie M. (1997). Effect of callipyge phenotype and cooking method on tenderness of several major lamb muscles. J. Anim. Sci..

